# Local-level spatiotemporal dynamics of COVID-19 transmission in the Greater Seoul Area, Korea: a view from a Bayesian perspective

**DOI:** 10.4178/epih.e2022016

**Published:** 2022-01-13

**Authors:** Youngbin Lym, Hyobin Lym, Ki-Jung Kim

**Affiliations:** 1 Research Institute of Natural Sciences, Chungnam National University, Daejeon Korea; 2 Center for Agricultural Outlook Sejong Office, Korea Rural Economic Institute, Cheongju, Korea; 3 Department of Smart Car Engineering, Doowon Technical University, Paju, Korea

**Keywords:** Coronavirus, Transmission risk, Public health, Local municipality

## Abstract

**OBJECTIVES:**

The purpose of this study was to enhance the understanding of the local-level spatiotemporal dynamics of COVID-19 transmission in the Greater Seoul Area (GSA), Korea, after its initial outbreak in January 2020.

**METHODS:**

Using the weekly aggregates of coronavirus disease 2019 (COVID-19) cases of 77 municipalities in the GSA, we examined the relative risks of COVID-19 infection across local districts over 50 consecutive weeks in 2020. To this end, we employed a spatiotemporal generalized linear mixed model under the hierarchical Bayesian framework. This allowed us to empirically examine the random effects of spatial alignments, temporal autocorrelation, and spatiotemporal interaction, along with fixed effects. Specifically, we utilized the conditional autoregressive and the weakly informative penalized complexity priors for hyperparameters of the random effects.

**RESULTS:**

Spatiotemporal interaction dominated the overall variability of random influences, followed by spatial correlation, whereas the temporal correlation appeared to be small. Considering these findings, we present dynamic changes in the spread of COVID-19 across local municipalities in the GSA as well as regions at elevated risk for further policy intervention.

**CONCLUSIONS:**

The outcomes of this study can contribute to advancing our understanding of the local-level COVID-19 spread dynamics within densely populated regions in Korea throughout 2020 from a different perspective, and will contribute to the development of regional safety planning against infectious diseases.

## GRAPHICAL ABSTRACT

[Fig f6-epih-44-e2022016]

## INTRODUCTION

As of December 31, 2020, more than 82 million coronavirus disease 2019 (COVID-19) cases were confirmed worldwide, with more than 1.8 million deaths attributed to this infectious disease [1]. According to the Korea Disease Control and Prevention Agency (KDCA), there were 60,740 confirmed cases (117 per 100,000 population) of COVID-19 and 900 deaths (1.58 per 100,000 population) in 2020 in Korea [2]. COVID-19 spread rapidly around the world due to the characteristics of respiratory infectious diseases, which are easily spread through direct or indirect contact with an infected person or through droplets from the infected [3]. In addition, some studies have pointed out that the spread of COVID-19 is caused by the growth in exchanges among regions following the development of transportation means and an increase in contact frequency due to high population density [4,5].

In 2020, there were 3 heterogeneous waves of the COVID-19 pandemic in Korea: the first in February–March, the second in August–September, and the third in mid-November. In response to each wave, the Korean government introduced and implemented several countermeasures against the disease (e.g., social distancing [SD], mandatory mask wearing, and tracing and tracking of confirmed patients) to suppress its spread without having a nationwide lockdown. We observed that throughout 2020, the pattern of COVID-19 outbreaks in Korea revealed unique features, which are assumed to be affected by the government’s response to the virus. We also acknowledge that each country has responded differently to COVID-19, leading to variation in the outcomes. This prompted us to investigate how COVID-19 spread across regions over time.

Numerous attempts have been made to clarify the association between the spread of COVID-19 spread and contributing factors, including the built environment, environmental conditions, air pollutions, and spatiotemporal dynamics. For instance, Briz-Redón & Serrano-Aroca [6] explored the early transmission dynamics of COVID-19 in Spain, considering the effects of temperature, space, and time. Briz-Redón [7] further examined the impact of modeling choices on the outcomes by comparing an array of spatiotemporal model specifications of 42 regions for 182 days in Catalonia, Spain. Kang et al. [8] explored the spatial dynamics of the COVID-19 outbreak in China at an early stage using Moran’s I, which revealed spatial associations among different regions. Valente & Laurini [9] estimated the spatial spread of mortality by COVID-19 on a global scale from late February 2020 to mid-February 2021, taking all 6 permanently inhabited continents into consideration. In an analysis of COVID-19 cases in Korea, Kim & Castro [10] implemented a retrospective space-time scan statistic to identify 12 spatiotemporal clusters in the early period of the pandemic. Lee et al. [11] recently attempted to clarify patterns of spatiotemporal changes and the diffusion of COVID-19 infections in 3 waves in Korea via ArcGIS and network analyses. The findings revealed disparities among the 3 waves of the pandemic in terms of transmission scale, speed, and direction. From a different perspective, Shim et al. [12] showed spatial variability in the reproduction number and doubling time of infections in Korea utilizing daily confirmed cases obtained from January to July 2020. Lee et al. [13] examined patterns in the daily reproduction number and fatalities, and then showed the spread pattern based upon a logistic growth model for the initial period of the pandemic. Lee et al. [14] studied the effect of 4 control measures (school closure, quarantine, isolation, and SD) on the transmission of COVID-19 via an extended age-structured susceptible-exposed-infectious-recovered model.

Although previous efforts have shown their relevance for understanding COVID-19 transmission across space over time, detailed local-level dynamics of diffusion (i.e., disease spread within a metropolitan region) have yet to be fully explored in Korea. Hence, motivated by these limitations of previous research endeavors, this study attempted to scrutinize spatiotemporal changes in the COVID-19 pandemic at the local municipality level in the Greater Seoul Area (GSA, i.e., the capital region of Korea) for 50 consecutive weeks of 2020. To elucidate the relative risks (RRs) of COVID-19 infection, we utilized a full hierarchical Bayesian approach under the generalized linear mixed modeling framework, which is flexible enough to account for the influences of unobserved heterogeneity originating from spatial alignments as well as temporal dependency. To this end, we argue that a systemic breakdown of latent effects provides an enhanced understanding of COVID-19 transmission over time within the GSA from a different perspective, contributing to evidence-based decision-making for regional safety planning against infectious diseases.

## MATERIALS AND METHODS

### Background

**Figure 1.**
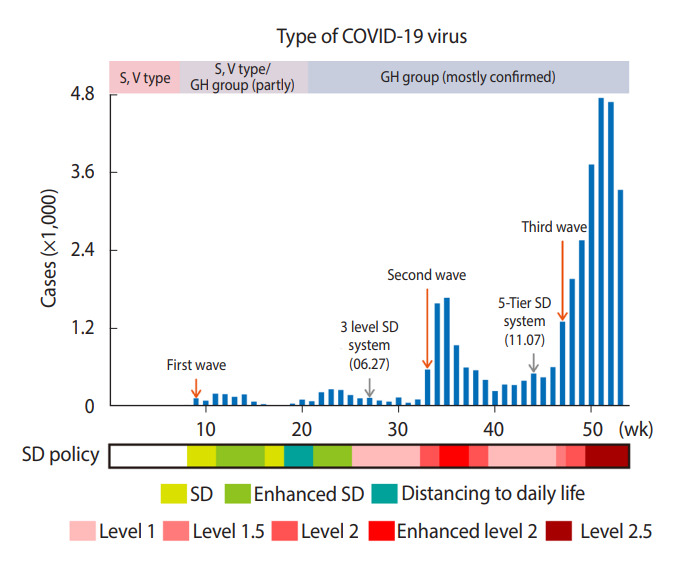
Year-round reported cases and public interventions (Greater Seoul Area). COVID-19, coronavirus disease 2019; SD, social distancing; S, clade S imported by inbound travelers (initial outbreak); V, clade V due to a large clade V cluster outbreak in Daegu-Gyeongbuk (earlier period); GH: clade GH type, predominant after S and V types, identified in Itaewon club and Coupang Logistics Center.

Prior to delving into the data in detail, we discuss the efforts of Korean public health authorities to keep COVID-19 under control. As shown in Figure 1, Korea has experienced 3 heterogeneous waves of COVID-19 between the initial outbreak in January and December 31, 2020.

The first wave started in Daegu, originating from a single source, the Shincheonji religious group, and at that time, the number of cumulative confirmed cases reached 5,213 [15]. After the first wave, the Korean government announced guidance for SD to prevent the local spread of COVID-19 by limiting contact among the public [16]. In accordance with the trajectory of the outbreak, SD was selectively adopted as “SD,” “enhanced SD,” and “distancing in daily life” [17]. Specifically, the government advised people to avoid gatherings as well as group activities, and to maintain safe distancing from February 29 to March 21 (the SD period). When sporadic infections occurred from March 22 to April 19 (the enhanced SD period), people were advised to stay at home, while facilities related to religion, indoor sports, and entertainment were closed [17]. In early May, there were a series of small group infections in the GSA (e.g., Itaewon club and Coupang Logistics Center) [12], but this was not classified as a pandemic. At the end of June, the Korean government introduced a systematized 3-level SD scheme corresponding to the severity of the outbreak (e.g., the number of daily cases for 2 weeks). This provided clear-cut public messaging and matching countermeasures [17].

The second wave of the pandemic was attributed to 2 origins: the Sarang Jeil church (1,170 cases) and a political rally on August 15 (650 cases). Unlike the first wave, the virus spread outside its initial sources, leading to a series of other small-cluster infections in related facilities and communities. As the spread intensified, the Korean government upgraded the “SD” stage to level 2 and even applied “enhanced” SD measure level 2 (temporarily), which imposed restrictions on the operations of restaurants and cafes. As a result, the number of confirmed cases fell below 100 in October, and the government lowered SD to level 1 (distancing in daily life). The KDCA—which was renamed and restructured from the Korea Centers for Disease Control and Prevention—announced a more finely tuned set of SD guidelines on November 7 that better reflected public health conditions on the ground [18]. The 3-level SD chart has been extended to a new 5-tier SD scheme (i.e., levels 1, 1.5, 2, 2.5, and 3). Each level is determined based on the average number of daily domestic infections over 1 week.

Meanwhile, the third wave started in the middle of November, heading into the winter season in which the virus is more active. This has posed greater challenges for controlling the pandemic due to a series of sporadic infections across local communities, making it harder to trace their transmission routes as compared to previous outbreaks. Moreover, amidst the third wave, the average number of daily confirmed cases reached 1,000 in December 2020.

### Data description of the study region

Approximately 59.7% of the total cumulative cases occurred in the GSA, followed by 36.3% in non-metropolitan areas and 4.0% in quarantine settings as of December 2020 in Korea. Daegu, the epicenter of the first wave, had the highest incidence rate of COVID-19 (320.17 per 100,000), followed by Seoul (195.24), and Gyeonggi-do (109.06) [2]. As previously mentioned, the first wave provoked by a church in Daegu did not spill over, and it was therefore suppressed shortly, whereas the spread of COVID-19 in the following waves showed different patterns within the GSA, challenging its control. This motivated us to explore the dynamic changes of COVID-19 transmission in the capital region, rather than in other places, with a specific focus on local-level transmission over time.

In that regard, 77 municipalities (i.e., *si/gun/gu*-level administrative divisions) within the GSA including Seoul, Gyeonggi-do, and Incheon, were selected as the spatial dimension of this study. For each municipality (district), we initially collected the number of daily confirmed cases of COVID-19 from January 24 (week 4) to December 31 (week 53), 2020. We then excluded cases of inbound travelers from foreign countries due to limitations of the data collected in earlier periods. To improve the validity and accuracy of our dataset, we cross-checked it with the confirmed cases of each municipality and updated the data by readjusting for reporting delays or errors. This ensured that we could obtain detailed daily time series of COVID-19 data. A weekly aggregate of the outbreak per municipality was utilized in subsequent investigations. Furthermore, we acquired geospatial data from OpenMarket at the National Spatial Data Infrastructure Portal [19] in order to relate those weekly aggregates and socio-demographic features to spatial data, which were prepared for the formal statistical assessment. Neighboring administrative units (i.e., municipalities) were defined when they shared a common border or boundary.

### Methodology

This study adopted a spatiotemporal generalized linear mixed model under a hierarchical Bayesian framework, which has been extensively utilized in the disease surveillance domain [20-22]. We attempted to estimate the RRs of disease occurrence among geographical units (*θ_it_*) through a statistical assessment. Formally,


(1) $$Data:\;Y_{it}|\lambda_{it}\;\sim \;Poisson(\lambda_{it}),\;\;\;\lambda_{it}\;=\;E_{it}\times \theta_{it}\;\;\;\;\;\;\;\;\;\;\;\;\;E_{it}\;=\;R_{t}^{\left(standardized\right)}P_{it}\;\;\;\;\;\;\;\;\;\;\;\;\;R_{t}^{\left(standardized\right)}=\frac{\sum_{i}^{}\;Y_{it}}{\sum_{i}^{}\;P_{it}}=\frac{total\;confirmed\;cases\;at\;time\;t}{sum\;of\;population\;of\;each\;district\;at\;time\;t}\;$$

(2) $$Process:\;\theta_{it}|\psi \sim \pi (\cdot |\psi ),\;\;\eta_{it}\;=\;log(\theta_{it})\;\;\;\;\;\;\;\;\;\;\;\;\;\eta_{it}\;=\;\beta_{0}+\sum_{j=1}^{p}\;\beta_{j}X_{i,j,t}+s_{i}+\gamma_{week(t)}+\gamma_{month(t)}+\delta_{it}\;\;\;\;\;\;\;\;\;\;\;\;\;s_{i}\sim BYM2\;specification\;(\phi \;\tau_{s}^{-1})\;\;\;\;\;\;\;\;\;\;\;\;\;\gamma_{week(t)}|\gamma_{week(t-1)}\sim Normal\;(\gamma_{week(t-1)},\;\tau_{week}^{-1})\;\;\;\;\;\;\;\;\;\;\;\;\;\gamma_{month(t)}|\gamma_{month(t-1)}\sim Normal\;(\gamma_{month(t-1)},\;\tau_{month}^{-1})\;\;\;\;\;\;\;\;\;\;\;\;\;\delta_{it}\sim Normal\;(0,\;\tau_{\delta }^{-1})$$

(3) $$Parameter:\;\psi \sim h(\cdot ),\;\psi ={\left(\beta_{0},\;\beta_{j},\;\tau_{week},\;\tau_{month},\;\tau_{\delta },\;\tau_{s},\;\phi \right)}^{T}\;\;\;\;\;\;\;\;\;\;\;\;\;\;\;\;\beta_{0}\sim Normal\;(0,\;10^{4})\;\;\;\;\;\;\;\;\;\;\;\;\;\;\;\;\beta_{j}\sim Normal\;(0,\;10^{4})\;for\;j=1,2,\cdots ,\;p\;\;\;\;\;\;\;\;\;\;\;\;\;\;\;\;\;pr((1/\sqrt{\tau_{week}})>1)=0.01\;\;\;\;\;\;\;\;\;\;\;\;\;\;\;\;pr((1/\sqrt{\tau_{month}})>1)=0.01\;\;\;\;\;\;\;\;\;\;\;\;\;\;\;\;pr((1/\sqrt{\tau_{\delta }})>1)=0.01\;\;\;\;\;\;\;\;\;\;\;\;\;\;\;\;pr((1/\sqrt{\tau_{s}})>(0.5/0.31))=0.01\;\;\;\;\;\;\;\;\;\;\;\;\;\;\;\;pr(\phi <0.5)=2/3\;$$

where the number of observed COVID-19 cases, *Y_it_*, for each municipality, *i*, on a week, *t*, is assumed to follow a Poisson distribution. The Poisson parameter, *λ_it_*, is further decomposed as the product of the expected value (or an offset), *E_it_* and the relative risk, *θ_it_*. Here, we used indirect standardization of *E_it_* to adjust for differences in exposure [20,22,23]. As shown in equation (1), *E_it_* is composed of $R_{t}^{\left(standardized\right)}$, the rate in the standard population (total number of cases divided by total population in all areas at time *t*) and *P_it_*, the population of area *Ii* at time *t*. The overall disease rate at time *t*, $R_{t}^{\left(standardized\right)}$, serves as a baseline that proportionately allocates the disease risk with respect to the local population. *η_it_* denotes a linear predictor, consisting of the fixed effects characterized by an intercept (*β*_0_) and the inner product between covariates (*X_i,j,t_*) and coefficients (*β_j_*), and of the random (latent) effects originating from spatial dependency (*s_i_*), non-linear weekly temporal trends (*γ_month(t)_*), non-linear monthly temporal influence (*γ_month(t)_*), and spatiotemporal interaction (*δ_it_*). Each *τ* stands for a precision parameter, an inverse of the variance. *ϕ* is a mixing parameter of the modified Besag-York-Mollié model (BYM2, a reparameterization of the BYM model) that is related to the marginal contribution of the scaled spatially structured random component [24].

As described, the RRs are systemically broken down into several components such as fixed and random effects. The random components are further decomposed into spatially correlated, temporally structured, and unstructured spatiotemporal effects. To consider spatial correlation, we employed a BYM2 model with the weakly informative penalized complexity (PC) prior [23,24]. For mathematical details, one may refer to Riebler et al. [25], Simpson et al. [23], and Fuglstad et al. [26]. Regarding temporal influences, we adopted non-linear dynamic trends characterized by the random walk of order one (RW1) process on both weekly and monthly shifts [27]. Finally, we assumed unstructured spatiotemporal interaction (Knorr-Held type I specification) [28], which captures unexplained variability in residuals after accounting for both fixed and random effects [22,27].

In defining PC priors for each random effect, we adopted the probability statement $Pr((1/\sqrt{\tau })>L)=a$ and Pr(ϕ<U)=b, where *L* and *U* are reasonable thresholds for parameters considered, while *a* and *b* are their corresponding probabilities. Based on this understanding, we present equation (3), which provides the detailed application of PC priors to the marginal precision parameters (*τ_week_*, *τ_month_*, *τ_s_*, *τ_δ_*) and the mixing parameter (*ϕ*).

### Ethics statement

Data utilized for this study are publicly available so that the ethical approval or consent from individuals is not required.

## RESULTS

### Model outcomes

Prior to delving into detailed results, it is worth noting that our formal assessment is based on the weekly aggregates of COVID-19 cases in each municipality from January 24 to December 31, 2020 (i.e., 77 municipalities× 50 weeks= 3,850 observations). To account for differences in exposure to COVID-19 infection, we relied upon the expected values as given in equation (1). We employed R-INLA, an efficient alternative to the traditional Markovchain Monte Carlo simulation for Bayesian models, to implement a series of Bayesian spatiotemporal models [29].

Table 1 shows the results of our optimal model based on goodness-of-fit measures such as the deviance information criterion and the widely applicable information criterion [30,31]. The size of individual municipalities and population density were considered as fixed effects in this study. The size of cities was estimated to have no important association with COVID-19 risks, as indicated by the large variability of the 95% credible intervals. Conversely, the influence of population density appeared to be positive, implying an elevated RR of COVID-19 infection.

**Figure 2.**
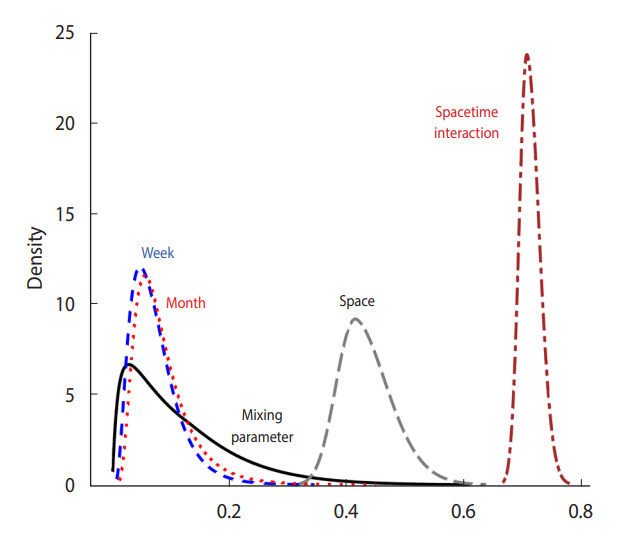
Marginal posterior distribution of hyperparameters.

Meanwhile, we present the posterior distribution of different types of unobserved heterogeneity in explaining random fluctuations in the RR of COVID-19. The posterior mean of the mixing parameter (*ϕ*) was estimated to be 0.117, implying that the marginal contribution of the scaled spatially structured random component was small, which reveals less spatial dependency of the risks (i.e., $\sqrt{0.117}=0.342$, the relative contribution of the scaled spatially structured random effect was 34.2% of the overall spatial influence, *s_i_*). Regarding temporal influences, we considered 2 RW1 priors for weekly and monthly effects. The higher precision of their respective hyperparameters (*τ_week_*, *τ_month_*) suggests that the temporal effects were relatively small. This is also depicted in Figure 2, which shows the posterior distribution of the standard deviation of the latent effects. Here, we transformed each precision parameter to the standard deviation parameter. In doing so, we employed the R-INLA function *inla.tmarginal()* to facilitate the conversion of parameters [23]. Moreover, random fluctuations by spatiotemporal interactions were identified, whose contribution to overall variability in the RR turned out to be the largest.

**Figure 3.**
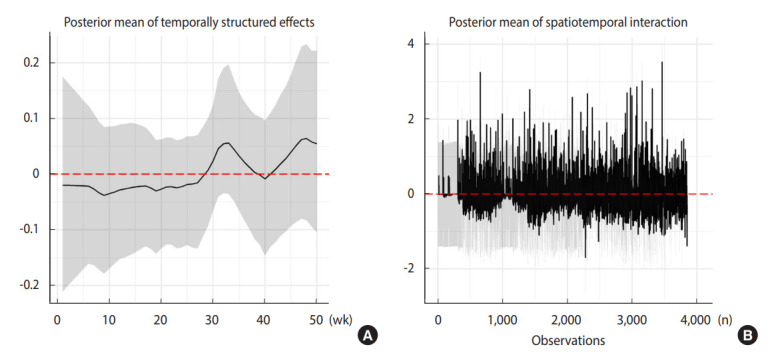
Latent influences from temporal dependence (A) and spatiotemporal interaction (B). (A) Posterior mean of temporally structured effects, (B) posterior mean of spatiotemporal interaction.

From a different perspective, we provided the marginal posterior mean and 95% credible intervals of temporal trends, as well as the spatiotemporal interactions, as shown in Figure 3. Figure 3A shows that the weekly time trend was not linear, supporting a non-parametric pattern (i.e., justifying our adoption of the RW1 process for temporally structured random effects). The marginal contribution of the temporal effect on the RRs was small, as the magnitude (value on the *y*-axis) indicates. In contrast, the influence of spatiotemporal interactions on the response seemed to be high (Figure 3B), capturing the unexplained residual variability of the COVID-19 RRs for municipalities over the study period.

### Spatiotemporal changes of relative risks

**Figure 4.**
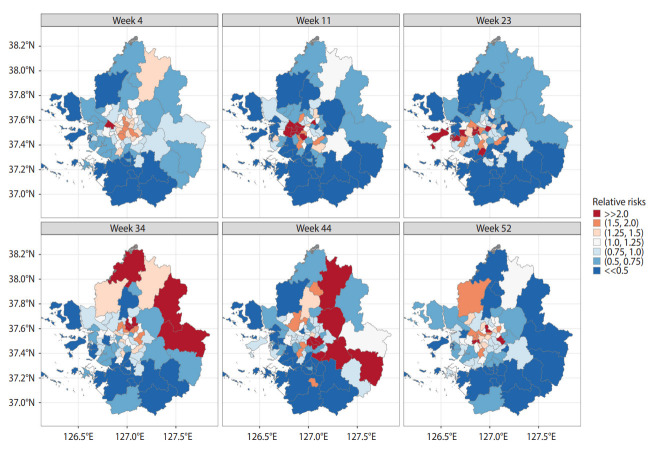
Spatiotemporal evolution of relative risks of COVID-19 in capital regions.

Considering these findings, we present Figure 4, which shows the spatiotemporal changes of the RRs across municipalities over the study period. Six temporal points were chosen considering representativeness in terms of seasonal variations and newly emerged clusters of COVID-19 infections. We selected week 4, as it was the first week that we confirmed positive COVID-19 cases in the GSA (winter), while week 11 and week 23 (spring) were included following the implementation of SD and enhanced SD policies. A surge of infectious cases was observed in week 34 (summer). We observed stabilizing trends in week 44 (fall) after the second wave. Finally, we witnessed a rebound attributed to winter cold, as people lowered their guard against COVID-19 infection, exhibited a lack of awareness, and engaged in frequent smaller gatherings in week 52 (winter).

Regarding the interpretation of the results, it is worth noting that when a municipality has a RR greater than 1, the city is regarded as an elevated risk region; conversely, a RR lower than 1 indicates lower risk. The pattern of the changes in RRs for each district shows that these changes were spatially and temporally structured, rather than random. For example, the estimated posterior mean suggests that municipalities in the northern and southern parts of Gyeonggi-do are likely to have been relatively safer than those in Seoul in week 4. In weeks 11 and 23, the patterns of municipalities neighboring Gyeonggi-do seemed to continue, while we observed newly emerged areas of higher risks in western Seoul (central regions of the GSA). In weeks 34 and 44, several districts with elevated COVID-19 risks emerged in northern Gyeonggi-do and in southern parts of Seoul. The disease map exhibits a different spatial distribution at week 52, reflecting changes in risk over space and time. In that regard, we verified that districts with similar RRs tended to be located together, implying some spatial dependency. The spatial pattern also changed with respect to temporal shifts, further revealing a temporal correlation. This suggests that accounting for random effects in the modeling process gives rise to an improved understanding of the RR of local-level COVID-19 transmission across the GSA.

**Figure 5.**
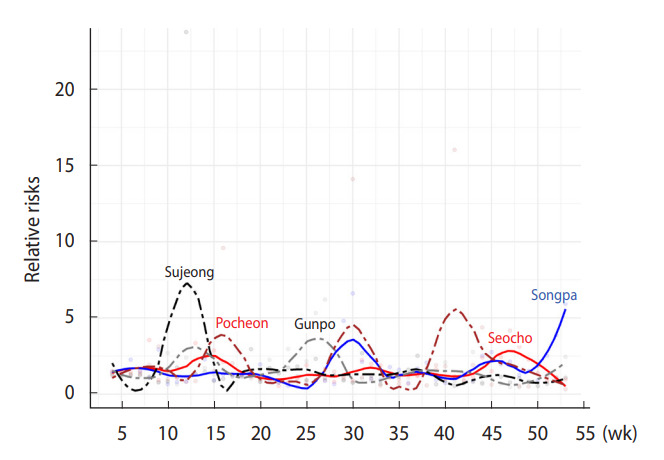
Evolution of relative risks in selected districts.

Meanwhile, we selected 5 districts (2 in Seoul and 3 in Gyeonggi-do) whose estimates of RRs tended to be higher than other regions, to show their individual patterns of change over time. The estimated posterior mean of the RRs was obtained for 50 weeks per municipality (i.e., 50 fitted values each). For each district, we applied locally estimated scatterplot smoothing regression to obtain a smoother curve (Figure 5), which helped us better appreciate the changing patterns of the risks. As shown in Figure 5, none of the districts had similar patterns in general but revealed individual specific dynamic changes of the RRs over time, reflecting the heterogeneous features of COVID-19 transmission. For example, a surge in Sujeong-gu occurred between week 10 and week 15, which was associated with religious gatherings, while the curve stabilized after this. Virus-prone facilities, such as gyms and saunas, were responsible for the surge in Seocho-gu, whereas a spike in Songpa-gu at week 53 originated from overcrowded correctional facilities, where highly transmissible viral infections can rapidly disseminate.

## DISCUSSION

This study explored the local-level spatiotemporal dynamics of COVID-19 spread within the capital region of Korea throughout 2020. For weekly accumulated cases per individual municipality over the study period (50 weeks in total), we considered the RRs of COVID-19 transmission as a point of interest and attempted to account for uncertainties associated with them. To this end, we utilized a generalized linear mixed modeling method under a spatiotemporal hierarchical Bayesian framework, which is flexible enough to accommodate latent influences from the neighboring structure of administrative units, their serial correlation, and spatiotemporal interactions on top of the fixed effects of covariates. We verified that the size of municipalities seemed to have no importance for the risk of COVID-19 infection, whereas more densely populated districts were likely to have an elevated risk. Regarding latent/random effects, our study reveals that spatiotemporal interaction dominated the overall variability of random influences, followed by spatial correlation, while temporal correlation appeared to be small. Based on these findings, we present the dynamic changes of COVID-19 spread across the GSA, which helped to clarify unique patterns of the disease as well as regions of elevated risk for further policy intervention.

In this study, however, there are several limitations that should be acknowledged. We present different policy (counter) measures against COVID-19 in Figure 1. Frequent changes as well as varied policy measures made variable construction rather challenging. Thus, while using a formal modeling process, we did not account for their potential impacts on mitigating the spread. There might have been lagged influences of policy measures on the spread of disease, which can be further examined in future research. In addition, numerous studies have investigated the associations of environmental factors (e.g., air pollution and climate), the built environment, and socio-demographics with COVID-19 transmission [32-35]. Unlike those previous efforts, which primarily focused on the direct effects of various influential factors, our study rather concentrated upon indirect/latent influences of space and time to explain the spatiotemporal dynamics of COVID-19 infections. To justify our approach, we based our study design upon Cressie [36], who suggested that spatial random effects can explain missing covariates and unobserved heterogeneity. Put differently, the adoption of our spatiotemporal random-effect model can accommodate influences from missing covariates such as policy measures, environmental factors, and socio-demographic features in the residual term.

Nonetheless, we argue that our findings can contribute to an enhanced understanding of local-level transmission mechanisms of COVID-19 in densely populated regions. Using the GSA in Korea as an example, our empirical study sheds light on the changing patterns of transmission of COVID-19 over time from a different perspective, complementing other works and supporting evidence-based policy decisions to combat infectious diseases (e.g., developing customized preventive policy measures considering spatial disparities in infection risk).

## Figures and Tables

**Figure f6-epih-44-e2022016:**
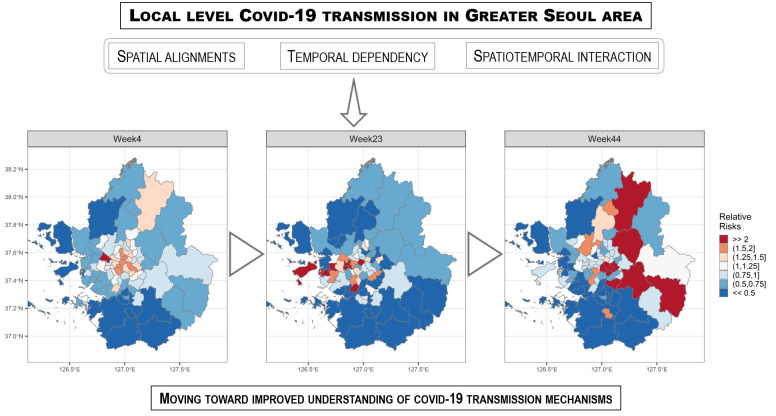


**Table 1.** Regression outcomes

Variables	Dependent variable: weekly aggregates of confirmed cases^1^				
	Mean±SD	2.5	50.0	97.5	Mode
Fixed effects					
Intercept	-1.931±0.338	-2.601	-1.930	-1.268	-1.928
Log (population density)	0.178±0.045	0.089	0.179	0.268	0.179
CitySizeLevel 2^2^	0.120±0.228	-0.326	0.119	0.570	0.117
CitySizeLevel 3^2^	-0.036±0.235	-0.498	-0.037	0.427	-0.038
CitySizeLevel 4^2^	0.068±0.254	-0.424	0.066	0.575	0.061
Random effects (hyperparameters)					
Precision of BYM2^3^	5.458±1.124	3.420	5.418	7.790	5.373
Mixing parameter of BYM2^3^	0.117±0.094	0.010	0.092	0.360	0.029
Precision of weekly effect^4^	476.949±892.856	30.490	230.350	2,474.950	75.895
Precision of monthly effect^4^	301.298±420.782	21.040	174.942	1,350.950	55.632
Precision of space-time interaction	1.969±0.094	1.780	1.973	2.140	1.992
Goodness-of-fit					
DIC	13,986.72	-	-	-	-
WAIC	13,780.51	-	-	-	-
Marginal log-likelihood	-8,121.01	-	-	-	-

SD, standard deviation; BYM2, a reparametrization of the Besag-York-Mollié model; DIC, deviance information criterion; WAIC, widely applicable information criterion.

1 Number of observations (N×T): 77 districts×50 weeks=3,850.

2 Reference category: CitySizeLevel1; In defining the size of each municipality, we considered the magnitude of the population (i.e., level 1 [<100 K], level 2 [<250 K], level 3 [<500 K], and level 4 [500 K+]).

3 There are 2 parameters (marginal precision and mixing parameter) in the BYM2 model.

4 The inverse of variance is a measure of precision (i.e., τ ^-1^=σ^2^); We considered both weekly and monthly structured temporal influences.
